# Mental and Behavioural Responses to Bahá’í Fasting: Looking behind the Scenes of a Religiously Motivated Intermittent Fast Using a Mixed Methods Approach

**DOI:** 10.3390/nu14051038

**Published:** 2022-02-28

**Authors:** Raphaela M. Ring, Clemens Eisenmann, Farid I. Kandil, Nico Steckhan, Sarah Demmrich, Caroline Klatte, Christian S. Kessler, Michael Jeitler, Michael Boschmann, Andreas Michalsen, Sarah B. Blakeslee, Barbara Stöckigt, Wiebke Stritter, Daniela A. Koppold-Liebscher

**Affiliations:** 1 Institute of Social Medicine, Epidemiology and Health Economics, Charité—Universitätsmedizin Berlin, Corporate Member of Freie Universität Berlin and Humboldt-Universität zu Berlin, 10117 Berlin, Germany; raphaela.ring@charite.de (R.M.R.); farid-ihab.kandil@charite.de (F.I.K.); nico.steckhan@charite.de (N.S.); caroline.klatte@charite.de (C.K.); christian.kessler@charite.de (C.S.K.); michael.jeitler@charite.de (M.J.); andreas.michalsen@charite.de (A.M.); barbara.stoeckigt@charite.de (B.S.); 2 Department of Sociology, University of Konstanz, 78457 Konstanz, Germany; clemens.eisenmann@uni-konstanz.de; 3 Connected Healthcare, Hasso Plattner Institute, University of Potsdam, 14482 Potsdam, Germany; 4 Department of Sociology/Cluster of Excellence “Religion & Politics”, University of Münster, 48143 Münster, Germany; kabogan@uni-muenster.de; 5 Department of Internal and Integrative Medicine, Immanuel Hospital Berlin, 10117 Berlin, Germany; 6 Experimental and Clinical Research Center (ECRC), a Cooperation between Charité—Universitätsmedizin Berlin and Max-Delbrück-Center for Molecular Medicine, 10117 Berlin, Germany; michael.boschmann@charite.de; 7 Charité—Universitätsmedizin Berlin, Corporate Member of Freie Universität Berlin, Humboldt Universität zu Berlin, and Berlin Institute of Health, 10117 Berlin, Germany; 8 Max-Delbrück-Center for Molecular Medicine (MDC), Helmholtz Association, 10117 Berlin, Germany; 9 Department of Pediatrics, Division of Oncology and Hematology, Charité—Universitätsmedizin Berlin, Freie Universität Berlin and Humboldt-Universität zu Berlin, 10117 Berlin, Germany; sarah.blakeslee@charite.de (S.B.B.); wiebke.stritter@charite.de (W.S.)

**Keywords:** intermittent food restriction, mindfulness, self-efficacy, well-being, mixed methods, health behaviour, coping ability, religiously motivated dry fasting

## Abstract

Background/Objective: Historically, fasting has been practiced not only for medical but also for religious reasons. Bahá’ís follow an annual religious intermittent dry fast of 19 days. We inquired into motivation behind and subjective health impacts of Bahá’í fasting. Methods: A convergent parallel mixed methods design was embedded in a clinical single arm observational study. Semi-structured individual interviews were conducted before (n = 7), during (n = 8), and after fasting (n = 8). Three months after the fasting period, two focus group interviews were conducted (n = 5/n = 3). A total of 146 Bahá’í volunteers answered an online survey at five time points before, during, and after fasting. Results: Fasting was found to play a central role for the religiosity of interviewees, implying changes in daily structures, spending time alone, engaging in religious practices, and experiencing social belonging. Results show an increase in mindfulness and well-being, which were accompanied by behavioural changes and experiences of self-efficacy and inner freedom. Survey scores point to an increase in mindfulness and well-being during fasting, while stress, anxiety, and fatigue decreased. Mindfulness remained elevated even three months after the fast. Conclusion: Bahá’í fasting seems to enhance participants’ mindfulness and well-being, lowering stress levels and reducing fatigue. Some of these effects lasted more than three months after fasting.

## 1. Introduction

Intermittent fasting (IF) and time-restricted eating (TRE) have gained increased awareness not only within the public community and media but also specifically within experimental and clinical research over the past decade [1]. Publications on IF and TRE mainly focus on anthropometric, physiological, biochemical, and immunological aspects of fasting [2,3,4]. In contrast, subjective experiences of fasting have received far less attention. This is surprising, as historically, periods of fasting have been recommended in almost all major religions worldwide [5,6,7]. Traditionally, religious fasting is not primarily seen as a specific dietary approach but as a tool for strengthening one’s will, getting closer to the divine, purifying one’s thoughts and elevating one’s consciousness [7,8]. In the clinical setting, these subjective experiences and related values of fasting might significantly determine its effects and outcomes and thus merit attention.

In this study, we approached fasting using individual qualitative and focus group interviews, combining them with quantitative questionnaire data to highlight subjective experiences associated with fasting through a multifaceted approach. We chose a traditional religious fasting that is similar to the widespread fasting of Ramadan yet allows for easier replicability in terms of seasonal and daily practice time-frames [5]. It also shares many characteristics with TRE, which has shown promising results regarding reduction in cardiovascular risk factors, obesity [9,10], and inflammatory markers, positively influencing gut microbiota and psychological well-being [1,11,12].

The Bahá’í Fast (BF) can be regarded as a diurnal intermittent dry fast where followers abstain from food and drink during daylight hours for 19 consecutive days every year in March. In general, the main beliefs in the Bahá’í Faith revolve around the concept of unity in diversity, which is reflected in the relative simplicity of rituals [13]. This can also be seen in BF, where no other laws or established traditions are connected to the fasting days, leaving the individual and the respective communities to decide freely about individual and social aspects of the fasting time. Bahá’ís have a highly diversified and at the same time highly structured community life, where international and national institutions, alongside local agencies, make communication with and between communities easy [14]. The community has a wide reach with approximately 8 million followers worldwide [15] and extending to more than 100,000 localities in virtually every country and territory worldwide [16]. Through this efficient structure for communication as well as the fact that Bahá’ís orientate themselves very closely on the original writings of Bahá’u’lláh, unity is highly valued in the community, and a homogeneity of practice can be supposed. Assuming from the above a relatively uniform practice of fasting within the religion, findings from Germany may be transferable to other places where BF is observed. In addition, the Bahá’í community has a high esteem for science and scientific research grounded in the original writings [17]. The resulting openness to science in the Bahá’í community encourages participation of members in studies on different aspects of religious life.

Fasting has been part of many religious traditions of the past and the present. Most studies have focused on Ramadan fasting, showing multiple physical and psychological effects [5,18,19,20,21,22]. There remains little research on Ramadan as a religious ritual from a medical perspective. Likewise, little medical research exists on Christian and Jewish fasting traditions, where most studies have been conducted on the Greek orthodox fast [23,24,25]. Sociological and anthropological studies on fasting have mainly been focused on its symbolic role, societal and political cohesion, or question of gender roles [26,27,28]. These studies emphasize the central role of rituals and eating cultures and further indicate a shift from fasting as an expression of piety to self-control [29,30]. However, apart from questions concerning clinical psychology, such as anorexia nervosa [31], medical dimensions of health, and subjective well-being have not been addressed.

In our previous publications on BF, we reported on the safety with no adverse changes in hydration indices observed. Effects found on our study population (n = 34) were a lowering of body mass index and total body fat mass as well as a reduction in blood glucose and HbA1c. The circadian phase estimated by transcript biomarkers of blood monocytes advanced by 1.1 h (n = 16) during fasting [32]. An analysis of some of the questionnaires addressing religious experiences in our sample showed an increase in religious experiences and mindfulness as well as a high sense of self-control/-efficacy during fasting [33].

In the study arm presented here, we aimed to examine the immediate and long-term effects of a religiously motivated, temporal restriction of food and fluid intake on subjective health and well-being. We wished to obtain insight not only on how fasting changes participants’ lives during fasting but also on how people feel about and prepare for the fast as well as to understand its effects. Furthermore, we explored the nuances in motivation and importance to individuals undertaking this kind of fasting. We chose a mixed methods approach to better detail the complexity of the phenomenon, combining outcomes of qualitative individual and focus group interviews with selected survey data.

## 2. Methods

### 2.1. Study Design

The study data presented in this paper were collected as part of a larger, clinical longitudinal, self-controlled, exploratory cohort study conducted at the Charité—Universitätsmedizin Berlin including among other approaches a convergent parallel mixed methods design. The outcomes of the clinical and laboratory findings have been reported elsewhere [32,34]. Questionnaire results specifying religious psychology and religious practice have also been published separately [33]. The institutional review board of Charité Universitätsmedizin Berlin (Charitéplatz 1, Berlin, Germany) approved the study protocol in January 2018 (ID: EA4/216/17). The study was registered with clinicaltrials.gov (ID: NCT03443739). Written informed consent was obtained from all participants prior to study entry. A detailed explanation of the main study design is found in our published study protocol [32].

Mixed methods combining quantitative and qualitative methods can increase understanding of complex issues and allow for the examination of findings from different vantage points (triangulation) [35]. They are especially suitable for health research, as using a variety of methods enables researchers to better understand the complexity of issues [36], which in our case included social factors, personal behaviours [35], and beliefs associated with religious fasting.

**Figure 1:**
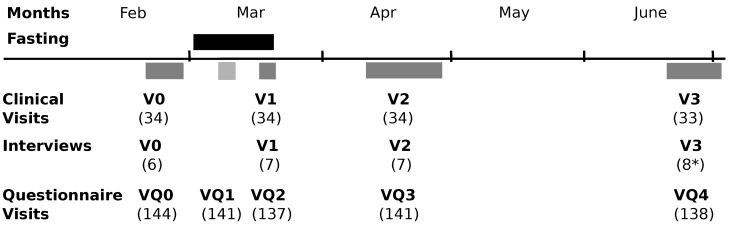
Timeline of visits. Timeline of data collection (V = clinical visit, VQ = questionnaires, (n) = number of participants at each visit, * for focus group interviews).

**Figure 2:**
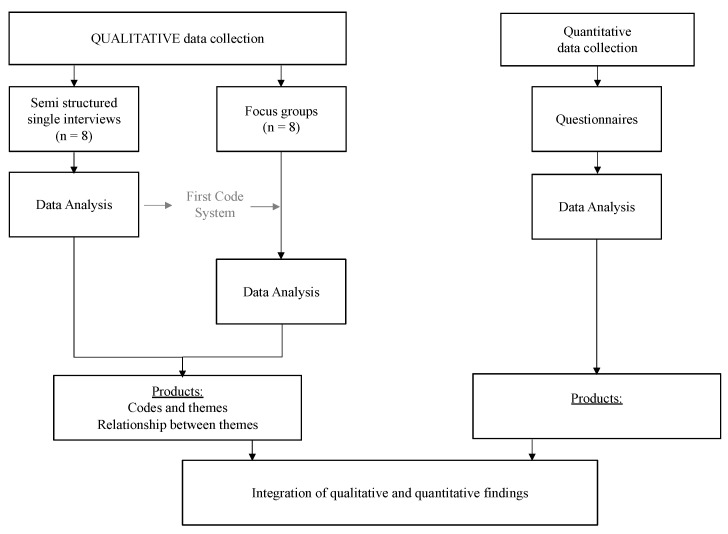
Study design.

In this study, we conducted questionnaires and interviews in parallel. Individual and focus group interviews were undertaken sequentially, the latter being implemented to verify the codes of the former. For an overview of the study timeline, please refer to Figure 1 The study design is illustrated in Figure 2, showing the different steps of data sampling, data analysis, and when integration of the results occurred. Data analysis was first conducted separately [36,37]; then, findings were integrated from the quantitative and qualitative portions by iteratively comparing and contrasting the data. This procedure of data collection and analysis was planned a priori, and the outcomes had no effect on this methodology.

### 2.2. Setting

The study was conducted at Charité—Universitätsmedizin Berlin. The interviews were conducted at the Department of Integrative Medicine at the Institute of Social Medicine, Epidemiology and Health Economics (IM) in a separate room that provided a quiet space for open discussion, and the questionnaires were distributed online via email. The timing of the visits is shown in Figure 1.

### 2.3. Participants

For this study, 172 healthy Bahá’í volunteers were screened. Of these, 146 were considered eligible for the survey on patient-reported outcomes and were enclosed between January and February 2018. Further details as well as eligibility criteria have been published elsewhere [32]. All eligible individuals filled out electronic questionnaires on subjective physical and psychological effects of Bahá’í Fasting. Of these, all participants living in the wider region of Berlin were invited to participate in laboratory tests, to which 34 subjects consented [32].

The interview sampling was chosen from this pool of participants. Individual interview and focus group sampling was based on the principle of maximum variation to balance gender and age. In accordance with theoretical sampling, the sample size was held flexibly and was iteratively determined by sample cohesion and saturation for the scope of this research [38,39]. First, individual interviews were undertaken with recruited participants to understand and explore concepts about fasting and religious practice on a personal level. After extracting our codes from these interviews, we invited participants to focus groups to investigate if and how the concepts found in the codes were discussed in a group setting. To avoid bias through influences of the semi-structured single-interviews and allow comparison between single-interviews and group discussions, we conducted one group discussion with interviewees who had participated in the single-interviews beforehand (focus group B) and one group discussion with respondents who had not yet participated in any interview (focus group A).

### 2.4. Qualitative Design

#### 2.4.1. Semi-Structured Individual Interviews

The interviews were generally timed according to when the participants came for the other study visits before, during, and three weeks after the fast. Where this was not possible, suitable individual arrangements were made. For the semi-structured interviews at the baseline visit (V0), we developed a guideline based on clinical experience and previous research findings, without determining possible outcomes beforehand. The interview guideline focused on the meaning of fasting for the interviewees and its impact on their daily life and their perception of stress during and after the fast. Moreover, it contained questions on their motivation to fast and whether there were other aspects than religious motivators, the meaning and importance of communal activities and support during the fast, difficulties with fasting, and changes in dietary or other habits during the fast.

As spirituality and religiosity are concepts that are being discussed intensively in qualitative research settings, they can be understood in different ways [40,41], which we did not aim to differentiate in our work. Instead, we decided to let the interviewee explain her or his concept of each category at the beginning of the first interview. By doing so, we ensured that the interviewer knew what the interviewee was talking about when using those terms and vice versa.

For the following two interview rounds (V1 and V2), we created an interview guide focused on topics emphasised by interviewees at baseline, which is a typical procedure for qualitative interviews [42,43,44]. Each interview was recorded with a recording device and was deleted after transcription. For interview guidelines, please refer to Supplementary File S1.

#### 2.4.2. Focus Groups

To validate the codes generated in the individual interviews, we conducted focus group interviews. Here, the group was encouraged to openly discuss the topics the participants chose to focus on [45,46,47]. The focus group interview guide encouraged open discussion in the group. The guide asked for a definition of fasting, including health aspects and food intake; they asked the participants to describe important aspects of fasting and asked the group about the difference between religious fasting and therapeutic fasting. Each interview was recorded with a recording device and was deleted after transcription. For the interview guide, see Supplementary File S1.

### 2.5. Quantitative Design

The survey was conducted at five time points: before fasting (VQ0), on the first (VQ1) and third (VQ2) weeks of the fast, as well as three weeks (VQ4) and three months (VQ5) later (for more details, please refer to Figure 2). Quantitative data collection was completed using electronic questionnaires using Limesurvey v2, running on virtualised machines on Charité’s servers. Questionnaires used the validated German translations of the Short Depression and Happiness Scale (SDHS) [48], Hospital Anxiety and Depression Scale (HADS) [49], Mindful Attention and Awareness Scale (MAAS) [50], Profile of Mood States (POMS) [51], WHO-5 [52], Cohens Stress Scale (CSS) [53], and a self-efficacy scale (ASKU) [54]. Additional questionnaires focusing on religious experience and spirituality together with elements of a self-developed questionnaire specific to this religious fast (Bahá’í fasting experience) have been published elsewhere [33].

### 2.6. Data Analysis

#### 2.6.1. Qualitative Data Analysis

All interviews were transcribed with f4transkript, then pseudonymised, openly and inductively coded within the software f4analyse, then condensed, summarised, or specified in order to find connections that would allow us to build categories following the principles of grounded theory [43]. Coding was completed by RR, discussed, and partly recoded by CE. Problems and conflicts in coding were discussed, and consensus was reached in regular meetings with further team members (NS and DL). The process of finding connections between single codes was characterised by consequent exchange between researchers about ideas on different passages and comparisons about the current results of quantitative and qualitative methods. Memos were written to reflect on the research process. Repeated connections between different codes appeared through an iterative process of axial coding whereby connections developed into our categories.

#### 2.6.2. Quantitative Data Analysis

The statistical analysis of the outcomes was an explorative one. All questionnaires were analysed using one-way repeated measures ANOVA with time as the fixed factor and subjects as random factors. Mauchley’s test was used to assess the assumption of sphericity. If needed, the common correction instruments of Greenhouse–Geisser and Huynh–Feldt epsilon were used. Analyses were based on an intention-to-treat approach using multiple imputation from the mice package. Post hoc multiple comparisons were completed using Student’s *t*-test with Benjamini–Hochberg adjustment. Then, 95% confidence intervals were calculated as well as omega-squared effect sizes. All statistical analyses were completed using the statistical programming language R version 4.

## 3. Results

Semi-structured individual interviews were conducted at three timepoints: before (n = 7), during (n = 8), and after fasting (n = 8). In total, 23 individual interviews were conducted. Three months after the fast, two focus group interviews were conducted to verify the codes developed from the individual interviews, with a total of n = 8 participants. A total of thirteen different participants were interviewed in the course of our study, either individually or in a group.

We interviewed four women and four men aged 21 to 69 (P1–P8) individually. The main occupational characteristics that describe the interviewees follow: two students, a person on long-term sick leave, a teacher, a psychotherapist, an artist (freelancer), one geriatric nurse in training, and an office employee. At the first study visit time point (V0), we interviewed seven participants, and at the second study time point (V1), one more person was interviewed to balance out age. Therefore, three interviews were conducted with seven interview partners, and two additional interviews were conducted with the 8th participant. Out of these 23 interviews, the three interviews of one interview partner were excluded from the analysis, because the interviewee struggled with language comprehension, and interviews became too long and hard to understand, leaving space for misunderstandings and wrong interpretations. Therefore, 20 single interviews were included in the analysis.

For focus group A, we recruited five interviewees, two women and three men, out of the pool of participants not having participated in the individual interviews. The main occupational characteristics that describe the focus group participants were an office employee, a former teacher, who had not been working for two years, two biologists, of whom one worked at a publishing house, and one chemist owning his own company. For focus group B, we recruited three former interviewees: two women and one man. Focus groups took place at time point V3.

Questionnaires were sent out to 146 participants and collected at five separate time points: VQ0, VQ1, VQ2, VQ3, and VQ4. A description of the baseline characteristics of participants can be seen in Table 1.

### 3.1. Qualitative Interview Findings

From the individual interviews (I), we extracted 42 codes, which were summarised in three main categories: (A) grounded in religion, (B) elements of fasting, and (C) impacts of fasting. For the verification of these codes, the two focus groups (II) were conducted. During analysis, theoretical saturation was reached for the main topics of interest through our qualitative exploration and iteratively validated in the incremental elicitation and analysis process.

 *(I).*  ***Individual interviews***

 *(A).*  ***Grounded in religion***

All interviewed Bahá’ís described fasting as deeply grounded in their religion. Therefore, religiosity is a pervasive dimension that we differentiated in two categories, “Trust in God” and “Meaning of religiosity for fasters”, and briefly summarise below. For an overview of these results, see Table 2.

Religiosity was described as an expression of love, trust in, and connection to God. Some interviewees understood spirituality as part of their religious belief, a mental attitude of how to connect with and perceive nature and others. The motivation and endurance to fast was described to be based on lived religiousness, which included an inner mindset, a will for personal growth, as well as a marked dedication to religious ordinances. All interviewees viewed their behaviour as a deference to God’s directive and an expression of trusting in God’s word and his support. One interviewee even stated that “*all religious commandments that He gives us as individuals and as society are good for us and lead us to progress (...). So, if He said to run up and down the stairs two at a time, I would do it!*” *(P7, I2, 123)*.

Three interviewees reported that submission to God’s will engendered the possibility to pass the responsibility over to God at the same time.

Religious laws were seen by all interviewees as an inherent part of Bahá’í religious life, and fasting was described as a central part of those. To follow religious laws was either seen as a willing and voluntary submission to God’s will or as a duty. “*I actually experience the scriptures as being the greatest support (...) and the providence that it (fasting) is a fixed component of Bahá’í life and included in these whole commandments is also this daily praying and meditating, which is intended throughout the whole year anyway, not only during the fasting period.*” *(P1, I1, section 78)*.

Interviewees described religious laws as an “operating instruction” for personal growth (P4, I3, 102). Religious fasting was described as “*a spiritual and religious exercise, leading you closer to God*” (P2, I2, 126). During the fast, interviewees emphasised focusing on their religion. Two-thirds reported feeling an improvement of their connection to God. The existence of the directive to fast was described as a supportive “*cornerstone for daily life*” (P5, I3, section 84).

*“I think too little about my religion during the year and during these 19 days, I think about it more. (...) I read more of the texts and can tell that it does me good…Like a homecoming.”*

(P2, I2, section 80)

 *(B).*  ***Elements of Fasting***

We identified four main prerequisites for the fasting period, as expressed by several interviewees: motivation, a sense of community, the opportunity to spend time alone, and a changed daily structure. Table 3 summarises these elements.

- Motivation

As participants expressed the belief in the benefit of God’s directives, they also expressed a high motivation to follow them. Repeating the fast annually was seen by all but one interviewee as reinforcing their described positive experiences, increasing their motivation to fast again. These experiences included physical well-being, a deepened feeling of religiousness and closeness to God, and an intensified feeling of connectedness with oneself. Additionally, some expressed the hope for a more conscious and healthier diet and a better-regulated eating behaviour. One person articulated the wish to lose weight, while another hoped for rejuvenating effects. Some of the interviewees mentioned feeling lighter in their body through fasting and hoped to reexperience this when fasting again. The outlook of an improved daily structure and seeing the fast as a chance to set goals for the upcoming year were also mentioned. Nevertheless, these elements were described as side effects that would not be important enough to fast were it not for the religious law.

- Changed daily structure

As eating and drinking is allowed only between sunset and sunrise, all interviewees described this as the most substantial influencing factor of day-to-day structure. They woke up early enough to eat and, of particular importance to all interviewees, prayed before eating. For all interviewees, fasting created a structure that they normally did not have, which was described as beneficial by all but one. How intensely an individual experienced these structural changes as different from their normal life differed from person to person. While one interviewee did “not have to change [much], I just have to get up a little earlier” (P1, I1, 81), another described that life was “*really totally different: nutritionally, sleepwise (…).*” *(P2, I2, 25).* Some interviewees explained that fasting helped them reduce distractions and use their time in a more systematic way. For six interviewees, it provided time to pray, read in the religious texts, and reflect on themselves, which was highly appreciated and valued during fasting. Challenges narrated by some interviewees were struggling to balance investment of time between the demands of the fast and other obligations. One interviewee struggled because of family life with young children, another because of homework for university. The latter reported suffering under the lack of time to focus on the fast while feeling pressured to work for university.

- Sense of community

Except for one interviewee, all reported spending more time with their religious community, friends, or family during the fast. While the two youngest interviewees focused mainly on their Bahá’í friends during fasting, the older participants used the fast to connect with a wider network of friends, family, colleagues, or neighbours. According to four interviewees, communication with Bahá’ís and non-Bahá’ís allowed for new perspectives on the religious texts and their lives. Three interviewees reported that intellectual exchange with other Bahá’ís deepened insights in their religion; for one, it expanded her consciousness. Four interviewees mentioned that interaction with others helped them become aware of their pronounced irritability or impatience during fasting. Realising these tendencies was described as helpful, as the respective interviewees saw it as a chance to consciously change their behaviour. In addition to these valued aspects of socialising during the fast, three interviewees emphasised how breaking their fast in the evening in company made them eat more and heavier food and go to bed later than compared with eating alone, which made the next day more challenging. In contrast, four interviewees reported beginning the day with a collective breakfast followed by shared prayers and discussions on religious texts, which was described as very supportive and enjoyable, facilitating early rising.

- Opportunity to spend time alone

“*It was very precious for me, at least the freedom to be alone (...) because I find that during the fasting period personal reflection is also very important (...). The introspection. And you cannot do that in company*” *(P1, I2, 122).* Meditating, praying, and reflecting on themselves and their lives were considered central elements of the Bahá’í fast by all interviewees. They reported changing their daily structure to make time to do so. While one of the two youngest interviewees did not profit from her time without the group at all and emphasised that she would have needed more time in community, the other one reported after the fast, that she had for the first time found out that time on her own was also very valuable. All other interviewees emphasised the importance of having time alone for self-reflection and prayers. One interviewee stated that he appreciated time in community but believes that “*the way to the search for God needs to happen in solitude*” *(P2, I2, 98).*

 *(C).*  ***Impacts of Fasting***

Interviewees reported that fasting changed their individual behaviour. They described bodily changes, increased well-being and mindfulness, the experience of discipline and freedom, as well as changes in daily habits. Table 4 illustrates these findings.

- Experiencing physical consequences of behavioural changes during fasting

The limited amount of time allowed for food and fluid intake during fasting was said to lead to a shift of attention to normally mundane, incidental tasks. All interviewees discovered effects of their behavioural changes on their bodies and their well-being. “*I think it really is this experience! This bodily experience of consciously restricting something during the day and feeling what it does within the body, and that it is actually good*” *(P1, I2, 132)*. Many interviewees enjoyed the feeling of an empty gut and physical lightness. Except for one interviewee, all reported drinking more than usual to avoid thirst during the daily fasting interval. Three interviewees emphasised how important it was that they went to bed early. During the fast, they discovered how lack of sleep had an intense negative impact on their physical condition. One interviewee linked the feeling of an empty, quiet stomach directly to feeling more energetic. Becoming aware of how their body felt while fasting heightened the perception of bodily changes. Interviewees reported employing different strategies to conserve their energy, e.g., moving more consciously and slower; being careful to avoid dizziness; working less or stopping earlier; and not talking much to avoid becoming thirsty.

- Improved well-being

All fasters reported being happy about fasting itself. Even though it was described as burdensome by one interviewee, still, he was glad that he had to focus on himself and his body and valued this experience. Fasting was perceived as a physical and spiritual cleansing by two interviewees. During the fast, three interviewees described feeling light and relaxed, which was caused by their focus on their religion, their increased introspection, and the physical sensation of an empty stomach. By not overeating, eating qualitatively good food, drinking more fluids than usual before and after breaking the fast, and sleeping enough, all but one reported feeling physically comfortable. One interviewee explained that only the fast reminded him of his body, which he stated not treating well enough in general. Although he reported suffering from lack of sleep, hunger, and tiredness during fasting, he perceived it as positive experience: “*It’s cool to fast, take a break, to tell the body: ‘now you’ll do something else (...)’ That’s good (...), you get out of your normal routine, your normal lifestyle, to step aside. And sometimes it hurts. But because it pushes you, gives you another pulse. That’s refreshing. That’s beneficial*” *(P2, I2, 130)*. Early mornings, before sunrise in particular, were often mentioned as a very special and comforting time of the day. Three interviewees underscored feeling more energetic than usual after managing the first days of fasting. Although all interviewees reported being happy about fasting, three were unsatisfied with their external life circumstances during fasting but stated they were pleased to fast because they were following the word of God.

- Mindfulness

Interviewees reported very different experiences of increased awareness of their body, their surroundings, and others. Three interviewees described benefits in the form of greater consciousness and awareness. By not eating, some interviewees mentioned they experienced freedom from physical, mundane needs: “*The whole body is subordinated to the spirit*” *(P7, I2, 61)*. One interviewee stressed this freedom as an important outcome of fasting. Others emphasised a more transcendent experience in the sense of a higher sensitivity towards nature and entering a dream state of mind while awake. Four interviewees described learning to let go of more than only hunger and thirst. “*This moment of letting go. That you notice how many things feel easier because of it. I think that also influences daily life, starting the day more relaxed.*” *(P1, I2, 74)*. Accordingly, five interviewees described fasting as “*a time where you become aware of what is central in life or where focus shifts on yourself.*” *(P3, I1, 43–44).* Two interviewees spoke extensively about finding one’s place in the world, a feeling described as being part of the larger order of the world, and saw fasting as a “*recalibration of life*” *(P7, I1, 48).* Three interviewees reported a heightened awareness of their feelings and emotions, which helped them either to react more consciously, to not react but observe and reflect, or to feel more relaxed in stressful situations. All but one interviewee reported experiencing self-sufficiency and an increasing sense of empathy, becoming kinder and more affectionate. Additionally, one interviewee mentioned a renewed sense of inner balance: “*I had this feeling of becoming one with myself again.*” *(P1, I1, 56).*

- Discipline and freedom

Challenges were reported by all interviewees, especially during the first days of fasting, starting by missing the meal at lunchtime. Four interviewees felt tired and one felt cold. Although the Bahá’í fast is a dry fast, thirst was seen as a challenge only by two interviewees, one of whom reported having a dry mouth when speaking too much. However, five interviewees mentioned irritability due to hunger and reported strategies they developed to face this challenge, for example “*just go to bed and wait for hunger to pass, sometimes*” *(P5, I1, 80)*. The sense of hunger was reported by three interviewees to decrease after the first few days. Experiencing the ability to apply the required discipline to manage these challenges was reported by all interviewees to be a decisive factor for persevering during difficult times of fasting. Feeling good while fasting strengthened resolve: “*If I can simply say* ‘*No, I can decide now’. Even if my body wants something else, then I am free.*” *(P5, I1, 33)*. This effect was reported by three interviewees as one of the longest lasting, most crucial consequences of fasting with the largest influence on their general life, as they learned to decide more freely.

- Changes in daily habits

During the fasting period, interviewees described measures they took to prepare for the fast, giving structure to their daily activities. Every interviewee had an individual way of preparation: two described a reduction in the amount of food intake one week before, another hid all her sweets, someone else planned his food shopping to avoid having to do a lot of it during the fast. One pre-cooked all meals for the whole fasting period, one planned which of the religious texts he would read during fasting. Two interviewees prepared mentally by increasing reflection on the upcoming fasting period. One interviewee reported having downloaded a prayer app on his smartphone to facilitate reading religious texts in the early mornings. Interviewees described starting preparations as early as January and as late as a few days before fasting. Just before the fast, there are a few days of Bahá’í festivities, which were valued as an important preparation by some interviewees. Interviewees reported changing different aspects of their eating behaviour during fasting. Every interviewee reported drinking more during fasting than before, and one person reported still drinking more even after the fasting period. All interviewees ate less. Some gave up sweets, chips, and other snacks during fasting and tried to make it last after the fasting period. One interviewee, who normally ate a lot of meat, increased the number of vegetables during fasting, another one focused on including more fruit in his diet. All but one interviewee reported enjoying food itself more, eating more slowly and consciously, and aimed to continue this after fasting. Only one interviewee described the change in his eating habits as difficult and not enjoyable.

Four of the interviewees wished to integrate their new eating habits into their daily life after the fast, including the amount of food intake and, for one interviewee, regular eating times. The aim to change behaviour long-lastingly was reported to be an annual response to fasting: “*Nineteen days is not long, but it seems to be long enough to establish new habits*” *(P7, I1, 173)*. Lasting effects varied individually, and most stated they did not expect being able to keep up all positive changes. Shortly after fasting, most interviewees still felt impacts of fasting on their behaviour. Changes in daily structure also allowed for more religious practices, which all wished to keep in their everyday lives. “*Before fasting everything was very normal in daily life, daily routine patterns (…) During fasting, about different elements of life, somehow more reflected and deliberate. (…) And afterwards is when you try to keep it, or some elements of it or so. I am still trying to tweak some small parts, I would say*.” *(P3, I3, 21)*.

 *(II).*  ***Focus groups***

*Focus group B* discussed fasting predominantly as a way of healing that is different from non-religious fasting because it includes not only physical but also spiritual aspects such as love and connection to God as an act of cleansing of the “outer” and “inner body”. Regarding changes experienced during fasting, focus group B talked extensively about drinking and eating habits. The main discussion points in this regard were not feeling a strong sense of thirst or hunger during the fast and enjoying eating less. They also discussed how changes in their eating habits continued beyond the fasting time.

*Focus group A* focused more on changes of their daily structure, describing fasting as an exercise of detachment and emphasising the importance of having time alone for reading in the holy scriptures, praying, and meditating. Mainly driven by one interviewee, focus group B discussed more about religious aspects of fasting than focus group A.

Both groups touched upon all main categories that were developed from the individual interviews. They talked about the importance of social support during the fast. The role of the family was emphasised, whose members may not be Bahá’ís but support the person fasting, in addition to the religious community. Experiencing discipline, challenges, and chances for personal growth were mentioned in both groups, such as increased well-being through fasting and the strengthened connection to God. Both groups discussed aspects of mindfulness enhanced by fasting, such as awareness and reflection of themselves and others, being more empathetic, and being able to let go of emotions: “*In the end it’s all a practice in letting go.*” *(Focus group A, 40)*. “*Through sacrifice of fundamentally different things in life, I gain greater consciousness.*” *(Focus group B, 6)*.

### 3.2. Quantitative Findings

**Figure 3:**
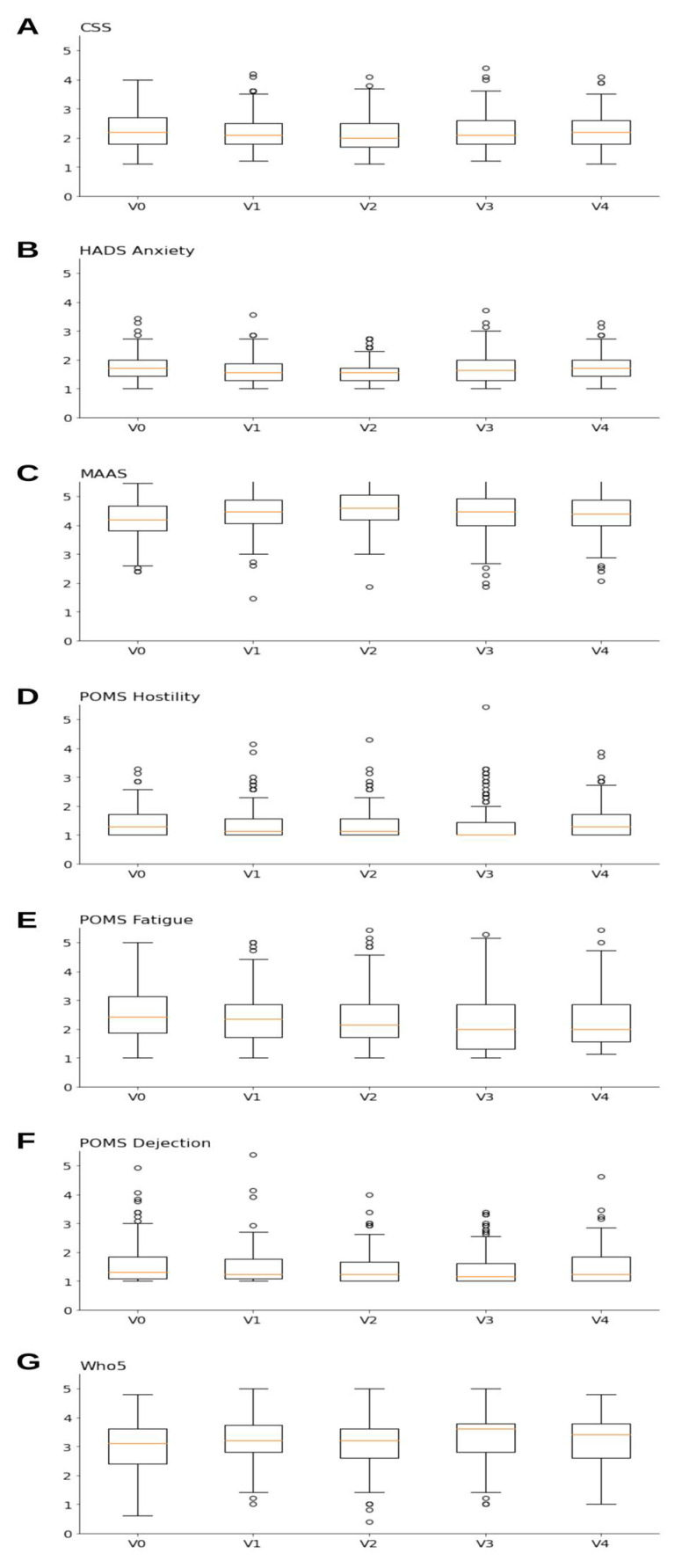
Box plots for questionnaires with *p*-values of 0.05 and below in the Friedman test: (**A**) CSS, (**B**) HADS, (**C**) MAAS, (**D**) POMS Hostility, (**E**) POMS Fatigue, (**F**) POMS Dejection and (**G**) WHO-5. Graphs present individual boxplots for each of the five visits (V0, V1, V2, V3, V4). Box boundaries represent 25% and 75% quantiles, the red line indicates the median, while the upper and lower whiskers show minimal and maximal values, except for outliers (“o”).

The positive effect on mindfulness was the most pronounced and sustained effect (*p* < 0.001). Quality of life improved during fasting (WHO5: *p* < 0.01), as did stress and anxiety symptoms (CSS-10: *p* < 0.01; HADS anxiety: *p* < 0.001). Measures of fatigue decreased during the fasting period (POMS Fatigue: *p* < 0.01; POMS Dejection: *p* < 0.01). No significant effects were identified for SDHS, HADS depression, self-efficacy, vigour (POMS vigour), and displeasure (POMS Hostility). For more details, please refer to Table 5 and Table 6 and the graphical illustration of the main statistical results in Figure 3.

### 3.3. Integration of Results

Summarising the results, interviews and questionnaires found increases in mindfulness and well-being (in the questionnaires MAAS and WHO-5 for the interviews codes “mindfulness” and “well-being”).

Stress and anxiety showed a reduction in the questionnaires CSS and HADS anxiety, while in the interviews, the codes “trust in God”, “specific social environment”, and “opportunity to spend time alone” show different aspects of attitudes and behaviour, which could be interpreted to be conducive to this reduction.

The ASKU questionnaire measuring self-efficacy showed high scores in our survey sample already at baseline and did not show significant changes during fasting. On the contrary, in the interviews, the topic of a rise in self-awareness and self-efficacy was mentioned under the codes “motivation” and “discipline and freedom”.

While in the interviews, certain “changes in daily structure” are reported, which could result in a rise in fatigue, the questionnaires showed a decrease in fatigue during fasting (POMS fatigue).

## 4. Discussion

To our knowledge, this is the first mixed methods approach to Bahá’í fasting, exploring a wide variety of effects on the physical, emotional, and mental state of followers. The changes in daily routines and the experience of fasting in general appears to cause an increase in mindfulness and well-being, along with having a mild anxiolytic and stress-reducing effect. The findings on self-efficacy, on the other hand, seem to vary between qualitative and quantitative findings.

Achieving higher levels of mindfulness through a dietary intervention such as fasting has not been previously described in the literature, to our knowledge. Mindfulness, embedded in meditation, was described by Kabat-Zinn as moment-by-moment awareness [55], while mindfulness as a mindset was described by Langer and Moldoveanu as a “process (…), which produces a greater sensitivity to one’s environment” [56]. We observed both of these characteristics of mindfulness in Bahá’í fasting. Fasting seems to potentially influence the fasters’ ability to recognise some somatic and psychological changes within themselves that enables a better connection to the outside world. Their ability to reflect, to let go, and accept the present moment rather than reacting to it is typical for mindfulness practices [55]. Interestingly, mindfulness appears to be increased without any obvious mindfulness intervention yet seems to have been encouraged through a focus on religion and religious practice. Aside from this, neurobiological effects could be responsible for some of these effects. Food restriction has been found to potentially increase sensitivity to and levels of serotonin [57,58,59], which has the potential to provoke transcendental experiences [58]. Choosing a mindful approach in stressful situations has also been shown to operate as a coping mechanism that enhances resilience and reduces stress [60,61,62].

The stress-reducing effects of mindfulness, in addition to the anti-depressive and anxiolytic effects [55], could partly explain our findings of increased well-being and reduced stress levels through fasting. The increased religiosity reported along with changes in health-related behaviour may have contributed to this effect. Well-being describes the cognitive and affective appraisal of one’s life. It has many aspects, covering physical, mental, emotional, social, and spiritual parts [63,64,65]. We found BF to target all these aspects. Participants reported feeling well physically with their changed daily habits during fasting, while the aspect of mindfulness touched on the mental level of well-being. They also mentioned various moments when they experienced social support or personal interactions during fasting that aided them mentally by knowing they were not fasting alone, thereby helping them to overcome challenges. One reason for Bahá’í fasting listed was that it brings about positive change in society through personal change towards a better version of oneself. Finding value in, and feeling valued by, society can promote an overall increase in social well-being [66]. Simply the belief that people will provide support with the needed resources by itself may lower the stress response [67]. A spiritual element of well-being was seen in our findings through the description of the personal, meaningful, and fulfilling relationship the participants had with God that was reinforced through the act of fasting [68]. Our findings are in line with findings on Ramadan fasting, as reported in a recent systematic review and meta-analysis on the subject that was found to have a positive influence on stress, anxiety, and depression [11]. The authors suggest lifestyle factors such as sleep deprivation as possible reasons for these effects [11]. Our findings suggest that one strong influence of personal well-being that may contribute to the observed psychological effects could be self-efficacy [69,70].

Regarding self-efficacy, our qualitative and quantitative findings appear to be contradictory. The fact that the ASKU questionnaire does not show any significant changes may be related to the high scores that our sample had at baseline. On the flip side, one explanation may be a lacking sensitivity of the questionnaire to changes occurring in a short period of time. The rise in self-efficacy reported in the interviews is in line with findings by Maniaci et al. describing improved self-efficacy through a fasting-mimicking diet [71]. Increased self-efficacy has been found to lower stress reaction [72,73,74], increase positive emotional affects, and lower anxiety [70]. Similar effects can also be found with the CSS, WHO-5, and HADS anxiety questionnaires, making it more likely that the ASKU did not have the sensitivity to show the changes in self-efficacy that we found reported in the interviews. Our interviewees expressed their heightened sense of self-efficacy as the ability to combat uneasiness due to hunger, sleep deprivation, or emotional disgruntlement by an inherent belief that they possessed the discipline to master the challenge of fasting. According to the transtheoretical model (Prochaska, Di Clemente, 1982), expected self-efficacy is found to grow with repetition of an action [69]. Through this mechanism, difficulties could potentially become progressively easier to manage with every subsequent fasting year. Most importantly, enhanced self-efficacy was reported in the interviews even after the conclusion of fasting and could explain the influence of stress response in daily life for a prolonged period of time. One of the strongest influences on self-efficacy is the first successful experience of managing a challenge, whereby practice and behavioural training are especially critical [69]. Since all of our participants reported having fasted at least one time before (see Table 1), this could help explain our sample’s high self-efficacy baseline score. This argument is supported by preliminary findings that show how previous fasting experience positively influences mood states in a current fasting situation [75]. The belief in one’s own self-efficacy expectation, which is the belief of having the competence to master a challenge, may have been strengthened by the interviewees’ trust in God. Certainly, the link between religion, self-efficacy, and fasting necessitates further research with a larger study population.

What is striking in our results is the very positive connotation that fasting seems to have for Bahá’ís. In the past, historical research on eating disorders has discussed religious rules as one possible point of origin [76], raising an awareness of the fine line between fasting and eating disorders in general. However, as for therapeutically prolonged fasting, no published findings have been found that support an induction of eating disorders through this practice [77,78]. One study in hospitalised fasting patients supports this notion, contrasting the feelings about and impact on eating and food abstention over time: the first few days were found to increase emotional fixation on food, while longer fasting periods led to a lack of appetite and fears regarding re-introduction of food [79]. Partial fasts, as observed in these preliminary findings with BF, may be found to be safer than prolonged fasts. Though not sufficiently studied, in a trial on a mainly Christian population in Finland, no association between religiosity and eating disorders could be found [80], while in a Muslim population in the United Arab Emirates, there seemed to be an association between the two [81]. It seems that the way religiosity is understood and lived makes the difference in the development of eating disorders [82]. In future trials, such aspects should be taken into account while exploring protective factors for eating disorders in different fasting regimen, be they religious or therapeutic.

Our study has limitations we would like to address: in the qualitative part of this study, to a large extent analysed by two researchers (RR and CE), there is the possibility of an incorrect interpretation. However, within the research team, we tried to address this problem by creating inter-reliability through constant exchange and discussions about codes, categories, and theory with CE, NS, WS, BS, and DL and merging the results with those of the questionnaires. We also made use of natural language processing to verify our codes, the results of which will be published in another paper. In the questionnaire survey, we used some clinical questionnaires on healthy individuals, not suffering from any clinically relevant condition. This limited sensitivity of the questionnaires for the study population resulted in high baseline scores and non-significant results in some of them. Additionally, a selection bias is probable for both methods due to the voluntary participation in the study as well as a social desirability bias, as our sample seems to show a high sense of religiosity. This possible bias must be given even more weight given the absence of a control group. On the other hand, the repetition of the interviews and questionnaires over a relatively long period of time allowed for a certain reliability of the measured outcomes. In addition, having contacted all registered Bahá’ís in Germany through the official national mailing list augmented the representability of the sample.

The long follow-up is a marked strength of this study, as is the synchronicity of sampling in both qualitative and quantitative methods. In addition, the use of focus group interviews to validate the codes stemming from the individual interviews and relatively large qualitative sample size add to the reliability of the data. This reliability is also reflected in the homogeneity of answers and the subsequent theoretical saturation of the interview outcomes.

The results presented here supported the interpretation of the laboratory and physiological data we collected in the sample. For example, we had expected to see at least a slight dehydration during fasting, as those fasting would not drink during daylight hours for 19 consecutive days. Surprisingly, we saw no dehydration, and on the contrary, some participants had a better hydration than at baseline. Interviewees during fasting reported drinking more fluids in the morning and evening and being more conscious about their hydration than usual, making a behavioural change a probable cause for our laboratory findings. In addition, the time-shift of -1.1 h on average seen in the circadian phase could be explained similarly, as most interviewees reported having a meal before sunrise, this being earlier than their normal breakfast. As meal timing can have a strong influence on circadian rhythms, this could have contributed to the observed shift.

Reflecting the outcomes reported in our paper on the religious aspects of BF [33], in light of the findings presented here, there might be an interesting link between difficulties experienced by fasters and their sense of achievement and freedom. Thus, it appears that the intensity of the challenges faced during fasting correlate with the outcomes in mindfulness and well-being. Interviewees reported that mastering the challenges presented by fasting gave them a sense of freedom. The link between the rise in mindfulness and well-being to the challenges of the fast could be the reaction to these difficulties as described by the interviewees.

## 5. Conclusions, Generalisability, and Future Work

Compared to Ramadan and other models of intermittent fasting, this type of fasting is relatively short and easy to undertake, and its safety was indicated by our laboratory findings [32]. BF seems to offer a chance to improve mindfulness, health-related behaviour, and subjective well-being. This said, further research about intermittent dry fasting in the Bahá’í religion and other religious contexts is necessary. Of interest would also be an examination of similar effects without a religious setting. Findings that may be reproduced in other fasting regimens, especially in intermittent fasting, could be of great benefit for a therapeutic supplement to such dietary approaches. With the growing popularity of intermittent fasting practice, the implications of supporting and enhancing social, motivational, and spiritual components should not be underestimated. From our findings, a predefined timeframe, time of year, collective experience, and daily timing of meals enhanced the subjective fasting experience and enabled the potential for changes in health-related habits. Rituals pertaining to eating culture in general and to fasting more specifically should be studied further to examine how they contribute to the positive effects described in the scientific literature so far. If further research supports these findings, an annual repeated 19 days intermittent dry fast such as BF could enrich medical and psychological therapeutic options, equipping patients with resources to enhance their overall health and well-being.

## Figures and Tables

**Table 1:** Baseline sociodemographic data of participants in questionnaire survey.

	n = 146 Mean (SD)
Age in years (mean (SD))	45.19 (13.85)
Sex = male (%)	65 (45.1)
Education (%)	
Still at school	0 (0.0)
Primary/secondary school graduate	4 (2.8)
Polytechnical secondary school graduate	1 (0.7)
Higher qualification secondary school graduate (Realschule)	4 (2.8)
High school graduate	34 (23.6)
Technical college or University graduate	95 (66.0)
Other	6 (4.2)
Gross wage/year (%)	
<20,000 Euro	60 (41.7)
20,000–40,000 Euros	30 (20.8)
40,000–60,000 Euros	19 (13.2)
60,000–80,000 Euros	14 (9.7)
>80,000 Euros	21 (14.6)
Fasting experience in the past (%)	
Yes, once	1 (0.7)
Yes, more than once	142 (98.6)
None	1 (0.7)
Kind of fasting experienced in the past (%)	
Prolonged therapeutic fasting	2 (1.4)
Religious fasting	138 (95.2)
Intermittent fasting	1 (0.7)
Other	2 (1.4)
Not specified	2 (1.4)
Duration of fasting experienced in the past (mean in days (SD))	18.64 (3.65)
Frequency of fasting in the past (%)	
Less than once a year	9 (6.3)
1–2 times per year	128 (90.1)
3–5 times per year	2 (1.4)
6–9 times per year	1 (0.7)
More than 10 times per year	2 (1.4)
Anticipated difficulties with fasting (%)	
Very easy	12 (8.3)
Easy	96 (66.7)
Difficult	34 (23.6)
Very difficult	2 (1.4)

**Table 2:** Grounded in religion.

Category	Code	Included in Code	Mentioned by Interviewee
Trust in God	Hand over responsibility to God	Feeling of the need to negotiate with God, only; justify actions towards God, find approvement in Gods’ word.	P7, P6, P5
	Submit to God	Submission to religious laws, importance of Gods’ word, obey to God, acceptance of limits due to religious laws.	P3, P6, P2
	Trust in God’s word	Certainty that everything will be all right, trust in God and religious acts, trust in religious laws and their benefit for oneself, God like a parent who explains and shows the world to his believers.	P1, P2, P3, P4, P5, P6, P7
	To find security in religion	Religious places as source of trust and security.	P1
Meaning of religiosity in life of fasting persons	Religious laws	Importance of religious laws, importance of a sense of duty by existence of religious laws, religious laws as challenge and gift, as a chance to experience something new, to learn, religious laws build identity and close up to the group to the outside.	P1, P2, P3, P4, P5, P6, P7
	Fasting means to align oneself with God	Heart is aligned to God, to come closer to God by the act of fasting, fasting is a religious act, a blessing, fasting is done for God.	P3, P1, P7, P5, P2
	Fasting is a central part of a religious life	Fasting is an element of religion, a fixed component, routine of Bahai life, fasting is spiritual.	P1, P7, P5
	To be Bahai means to aim for progress	Process of progress, maturity process, self-development.	P1, P7, P4, P2
	To do something good for the society	Support Gods’ project of development of the human being, change the society, being a critical mass, impulse for improvement of the world, to do good for society, positive influence on non-Bahaians.	P1, P7, P2
	To eat mindfully	To take time for eating, to eat and drink with more awareness, to be aware of the act of eating.	P3, P1, P7, P4, P6, P5, P2

Each code comes with an explanation under which premises it was used.

**Table 3:** Elements of fasting.

Category	Code	Included in Code	Mentioned by Interviewee
Motivation	Motivation	Earlier experiences that motivate to fast again, wish to return to God, to return to the core, wish to follow religious laws, wish to treat oneself by renunciation.	P1, P7, P4, P6, P5, P2
	Expectations	Description of concrete expectations from the fasting period.	P3, P1, P7, P4, P5
Changed daily structure	Structuring the day	Comparison of daily structure during fasting and daily life without fasting, descriptions of more or different structures, time efficiency, description of a fasting routine, flexibility of daily structure.	P3, P1, P7, P4, P6, P5, P2
	Traditions	Traditions, taken over from parents and self-made traditions.	P1, P7, P6, P2
	Intensify religious practices	Deepening of religious acts, spending more time with religious texts, meditation, and prayers.	P3, P1, P7, P6, P2, P5
Sense of community	Religious meetings are a source of well-being	Positive descriptions of religious get-togethers, community life during fasting, support by religious meetings.	P3, P1, P5
	Social support	Meaning of social support in general, support by family, friends, religious community, different importance of social groups.	P3, P1, P7, P4, P6, P5, P2
	Influence of community life on lent	Benefits and disadvantages of community life during lent.	P3, P1, P7, P4, P6, P5, P2
	Exchange with others influences the faster	Description of influences of conversations and interactions with people during lent.	P7, P4, P6, P5
Opportunity to spend time alone	To have time on my own	Descriptions of moments alone, values and importance of that time.	P3, P1, P7, P4, P6, P5, P2
	To eat mindfully	To take time for eating, to eat and drink with more awareness, to be aware of the act of eating.	P3, P1, P7, P4, P6, P5, P2

Each code comes with an explanation under which premises it was used.

**Table 4:** Impacts of fasting.

Category	Code	Included in Code	Mentioned by Interviewee
Experiencing physical consequences of behavioural changes	To get to know myself better	Impact of actions and experiences on oneself.	P3, P1, P7, P4, P6, P5
	To experience what is good for my body	Concrete actions that impact the body.	P3, P1, P7, P4, P6, P5, P2
Improved well-being	To influence well-being	Descriptions of joy, peace, mental and inner strengthening, pleasure, less concerns, feeling better, more balanced, contentment.	P3, P1, P7, P4, P6, P5
	Doing good to myself	Descriptions of concrete acts, where interviewees want to do something good to themselves, fasting as anti-depressive, fasting as treat.	P3, P1, P7, P4, P6, P5, P2
	To value fasting as positive	Positive descriptions of the value of fasting, gratitude for the ability to fast, fasting as inspiration.	P1, P7, P5, P2
	Energy	Higher levels of energy during the fast.	P1, P7, P6
	Lightness	Feeling of physical lightness, lightness as a new sense of vitality during fasting.	P1, P7, P4, P6
	Cleanse	Feeling of physical and mental cleanse.	P3, P1
Mindfulness	Feeling of integration into the world	A feeling of order, classified as a feeling of being part of the world, recalibration.	P4, P7
	Mindfulness	Letting go, not reacting, distancing oneself, seeing clearly, reported mindfulness, meditative actions, special sensations (feeling grounded, feeling of lightness).	P1, P4, P6, P5
	Higher awareness	Awareness, consciously doing something, feeling more conscious.	P3, P1, P4
	Focus changes	Focus and concentration on myself, focus on the central in life.	P1, P7, P4, P6, P5
	Being more sensitive and empathetic	Being more sensitive, empathetic, forgiving, friendly, loving, open to others.	P3, P1, P7, P4, P6, P5
	Reflecting over myself and life	Reflections about life, small things, feelings, self-reflection.	P3, P1, P5
	Overcoming the mundane	Body submits to mind, decisions free of physical needs, of constraints of nature, of the mundane, a state of dreaming while awake.	P3, P7, P5, P2
	Connectedness	Connectedness with God, with others, with oneself, with nature.	P3, P1, P7, P4, P6, P5
Discipline and freedom	Freedom	Feeling free, gaining freedom.	P7, P4, P5
	Challenges	Challenges experienced during fasting.	P3, P1, P7, P4, P6, P5, P2
	Discipline	Discipline.	P3, P1, P7, P4, P6, P5, P2
	Development	Development.	P3, P1, P4, P6, P5, P2
Changes in individual behaviour	Assistive preparations for fasting	Description of concrete preparations for the fasting period, acts, and mental preparations.	P3, P1, P7, P4, P6, P5, P2
	To create new habits	Descriptions of habits that are special during fasting and habits, which last after the fast.	P3, P1, P7, P4, P6, P5, P2
	To eat mindfully	To take time for eating, to eat and drink with more awareness, to be aware of the act of eating.	P3, P1, P7, P4, P6, P5, P2

Each code comes with an explanation under which premises it was used.

**Table 5:** Questionnaire results.

	Friedman Test		V0–V1		V0–V2		V0–V3		V0–V4	
Questionnaire	F	*p*	W	*p*	W	*p*	W	*p*	W	*p*
ASKU	0.0091	0.2569	1976.5	0.6452	1479.0	0.0101	1767.5	0.1473	2157.0	0.4289
CSS	0.0305	0.0014	3227.5	0.0058	2482.5	<0.0001	3812.0	0.066	4157.5	0.5039
HADS Anxiety	0.0873	<0.0001	1456.5	<0.0001	1096.5	<0.0001	2451.5	0.003	2793.0	0.0537
HADS Depression	0.0104	0.195	2656.0	0.4687	1877.5	0.0055	3180.0	0.3745	2625.0	0.8626
MAAS	0.0951	<0.0001	2862.5	<0.0001	1927.0	<0.0001	3065.0	0.0002	3297.0	0.0015
POMS Hostility	0.0288	0.0021	2287.0	0.1504	2293.0	0.087	2116.5	0.0232	3487.5	0.9517
POMS Fatigue	0.047	<0.0001	3818.0	0.0278	3727.0	0.0432	2604.0	<0.0001	3593.5	0.015
POMS Dejection	0.0415	<0.0001	2378.0	0.0076	1964.5	0.0002	2236.5	0.0006	3440.0	0.4263
POMS Vigour	0.004	0.6783	4743.5	0.5903	4692.0	0.7167	4294.5	0.2873	4438.5	0.7401
SDHS	0.0098	0.2211	2774.0	0.1538	1855.5	0.003	2675.0	0.0473	3087.0	0.2549
Who5	0.035	0.0004	3384.5	0.0081	3400.0	0.1099	2286.0	<0.0001	3339.5	0.0328

Results of the Friedman test across all 5 visits (F and *p*-values) and results of the fourWilcoxon tests applied between V0 and V1, V2, V3, and V4, respectively, as post-hoc tests (W and *p*-values). F = F-test of overall significance, p = *p*-value, W = W-value of Wilcoxon test statistic.

**Table 6:** Questionnaire results.

Questionnaire	Visit	M	SD	Med	Min	Q25%	Q75%	Max
ASKU	VO	4.02	0.67	4.00	1.00	3.67	4.33	5.00
	V1	4.03	0.72	4.00	2.00	3.67	4.67	5.00
	V2	4.13	0.68	4.00	1.00	3.67	4.67	5.00
	V3	4.08	0.66	4.00	1.00	4.00	4.33	5.00
	V4	4.04	0.67	4.00	1.67	3.67	4.67	5.00
CSS	VO	2.32	0.62	2.20	1.10	1.80	2.70	4.00
	V1	2.20	0.61	2.10	1.20	1.80	2.50	4.20
	V2	2.14	0.63	2.00	1.10	1.70	2.50	4.10
	V3	2.24	0.63	2.10	1.20	1.80	2.60	4.40
	V4	2.27	0.63	2.20	1.10	1.80	2.60	4.10
HADS Anxiety	VO	1.80	0.46	1.71	1.00	1.43	2.00	3.43
	V1	1.63	0.44	1.57	1.00	1.29	1.86	3.57
	V2	1.59	0.40	1.57	1.00	1.29	1.71	2.71
	V3	1.72	0.52	1.64	1.00	1.29	2.00	3.71
	V4	1.74	0.47	1.71	1.00	1.43	2.00	3.29
HADS Depression	VO	1.53	0.41	1.43	1.00	1.18	1.71	3.29
	V1	1.50	0.37	1.43	1.00	1.18	1.71	2.86
	V2	1.47	0.37	1.43	1.00	1.14	1.71	2.57
	V3	1.48	0.39	1.43	1.00	1.14	1.71	2.57
	V4	1.51	0.39	1.43	1.00	1.14	1.71	3.00
MAAS	VO	4.23	0.66	4.20	2.40	3.82	4.67	5.47
	V1	4.42	0.67	4.47	1.47	4.07	4.87	6.00
	V2	4.57	0.68	4.60	1.87	4.20	5.05	5.93
	V3	4.42	0.75	4.47	1.87	4.00	4.93	6.00
	V4	4.39	0.73	4.40	2.07	4.00	4.87	6.00
POMS Hostility	VO	1.45	0.52	1.29	1.00	1.00	1.71	3.29
	V1	1.39	0.56	1.14	1.00	1.00	1.57	4.14
	V2	1.37	0.55	1.14	1.00	1.00	1.57	4.29
	V3	1.38	0.66	1.00	1.00	1.00	1.43	5.43
	V4	1.45	0.59	1.29	1.00	1.00	1.71	3.86
POMS Fatigue	VO	2.53	0.91	2.43	1.00	1.86	3.14	5.00
	V1	2.38	0.88	2.36	1.00	1.71	2.86	5.00
	V2	2.36	0.92	2.14	1.00	1.71	2.86	5.43
	V3	2.17	0.92	2.00	1.00	1.32	2.86	5.29
	V4	2.32	0.96	2.00	1.14	1.57	2.86	5.43
POMS Dejection	VO	1.59	0.73	1.31	1.00	1.08	1.85	4.92
	V1	1.48	0.66	1.23	1.00	1.08	1.77	5.38
	V2	1.41	0.55	1.23	1.00	1.00	1.67	4.00
	V3	1.42	0.57	1.15	1.00	1.00	1.62	3.38
	V4	1.54	0.65	1.23	1.00	1.00	1.85	4.62
POMS Vigour	VO	3.31	0.91	3.29	1.57	2.57	3.86	6.00
	V1	3.28	0.93	3.14	1.29	2.57	4.00	5.86
	V2	3.35	0.98	3.14	1.57	2.71	4.00	6.00
	V3	3.40	0.97	3.29	1.14	2.71	4.11	5.57
	V4	3.35	0.98	3.29	1.00	2.57	4.00	5.57
SDHS	VO	2.34	0.50	2.50	0.50	2.00	2.67	3.00
	V1	2.39	0.50	2.50	1.00	2.17	2.83	3.00
	V2	2.44	0.48	2.50	0.50	2.17	2.83	3.00
	V3	2.43	0.47	2.50	1.00	2.04	2.83	3.00
	V4	2.40	0.53	2.50	0.67	2.00	2.83	3.00
Who5	VO	2.98	0.84	3.10	0.60	2.40	3.60	4.80
	V1	3.20	0.76	3.20	1.00	2.80	3.75	5.00
	V2	3.09	0.85	3.20	0.40	2.60	3.60	5.00
	V3	3.29	0.81	3.60	1.00	2.80	3.80	5.00
	V4	3.18	0.83	3.40	1.00	2.60	3.80	4.80

Descriptive statistics with mean (M), standard deviation (SD), median (Med), minimum (Min), maximum (Max) and 25 and 75 percent quantile (Q25%, Q75%) for each questionnaire and each visit.

## Data Availability

Data described in the manuscript will be made available upon request pending application and approval.

## Supplementary Materials

The following supporting information can be downloaded at: https://www.mdpi.com/article/10.3390/nu14051038/s1, File S1: Interview guide.

## Abbreviations

BF	Bahá’í Fasting
IM	Department of Integrative Medicine at the Institute of Social Medicine, Epidemiology and Health Economics
