# Supplementary material for: Mental and Behavioural Responses to Bahá’í Fasting: Looking behind the Scenes of a Religiously Motivated Intermittent Fast Using a Mixed Methods Approach
Source: Nutrients. 2022 Feb 28;14(5):1038. doi: 10.3390/nu14051038 (PMC8912886; doi:10.3390/nu14051038)
Supplement: Supplementary file 1 [file nutrients-14-01038-s001.zip › nutrients-1558319-supplementary.pdf]

Interview guide 1

- How long have you been a member of the Bahá'í community?
  Are you a converted member? --> What motivated you to do so? // Born Bahá'í.

- You are a practicing member of the Bahá'í community. Can you describe what makes you a Bahá'í / What does this mean to you? (Attitudes, organization of daily life, etc., important rituals, community activities).
- Would you describe yourself as a spiritual person?
- Can you describe spirituality to me in more detail? What does spiritual mean to you?
- What is spiritual about fasting? OR Why would you not describe your religion as spiritual? What differences do you see?
- Can you generally explain to me what fasting means to a Bahá'í?
- Are there any other aspects of fasting important to you?

  healthier // spending time on myself // spirituality community - where does the expectation come from // connection to God - where does the expectation come from // No.

- When was the last time you fasted?
  (yes, at XX) what was your experience like? (Physical/body feeling, spiritual, if applicable, during / after the fast) Please describe in more detail. What terms come up? Please explain. How is this evaluated positively / negatively?

- *(Never)* How are you preparing right now for this? Do you have support?

- What is especially important to you in preparing for the fast?
- What/ Are there adjustments to your daily life that are necessary to accommodate fasting?
- When you break your fast in the evening, How do you plan to eat? The same as you always do or do you eat differently during the fast than you normally do? What do you change and why?
- (*--> Mention pure rule-following if necessary*): How do you find fasting easy? Are there any problems?
- When you think about the upcoming fasting time: What does it trigger for you?
- What expectations do you have for fasting?
- What do you think - how do you think you will manage to cope with the fast?
- Is there anything else you would like to add?

Interview guide 2

- How are you? And how have you been the last few days? - Are there any changes in the way you feel?
- Was anything different than usual? How is that?
- Has your perception of yourself changed?
- Have you experienced anything special in the last two weeks that you might not have experienced without fasting? - What? What did it feel like?
- Did you notice any changes in yourself over the past two weeks? - What changes were they? How do you explain them?
  How has your body felt in the last two weeks compared to before?
- Do you currently feel like your body is getting what it needs? - What is missing?
- What are you eating right now? Is there anything new in your diet that you didn't eat or drink before fasting?

- What is religious about fasting?
- Have there been any experiences in the last few days that you can use to explain the spiritual aspect of fasting?
- How do you perceive your surroundings at the moment?
- Are you as focused as usual at the moment? What has changed?
- Is there any you just described to me that you have experienced previously during fasting?

- What expectations did you have in connection with the fast? And what expectations do you have of fasting right now?
- To what extent have these expectations been fulfilled so far?
- How satisfied are you with the fast so far?
- How satisfied are you so far with how you are practicing the fast? -- Is there anything you would want to change in the coming day or that you see in others that would make fasting easier for you.
- Have there been any challenging moments? How did you handle them?

- Comparing no-fasting to fasting, do you feel like you have a more structured day? What routines and habits are important to you during the fast?
- How does your day as a fasting person compare to a day of someone who is not fasting?
- Why do you fast?
- Can you explain what motivates you to fast? / For whom are you fasting? If you were to motivate someone else to fast, what would you say?

- Do you fast alone or do you fast together? → Together means in direct contact with other Bahá'í members, family or friends.
- When you fast, do you feel more connected to the people around you than usual? With whom are you more connected than usual?
- To what extent is this due to fasting?
- Who supports you in fasting? Who do you support when fasting? Why is this important? / What support is not important during this time?
- How do you feel when you look back on the past two weeks?
- Is there anything you wish you could keep for the long term that has changed now? What is it? Why?

- Is there anything I've forgotten? Is there anything else you'd like to add?

Interview guide 3

- Could you please describe the first day you stopped fasting.
  What did he / she do, how did he / she feel, with whom did he / she spend it? alone/together - why? Was something different?
- If we were to talk for the first time today about the past time before, during, and after fasting, how would you summarize it?

- Can you describe to me how you feel now compared to during the fast?
- What did you learn during the fast, what insights did you gain?
- How much time did you have in the last three weeks for things that are important to you? -- *Are these activities/people etc. that were also important to you during the fast?*
- How was it during the fast with these activities/people? *More/less time?*

- Are you now reintegrated into what you would describe as your normal daily life? *If not:* what is different? How does this happen? *Inquire about changes*
- To what extent would you describe your everyday life as stressful? Were there been any changes during the fast?
- Did the fast have any other impact / How did the fast impact your daily life? *Structure? Diet?*
- How do you feel about this everyday life? Did you feel different during the fast?

- Could you describe how much you have been eating and drinking since you stopped fasting compared to before?
- Are there any changes to the foods you buy? *Are they the same quality as before?*
- How did you experience shopping itself after fasting? Is it still the same?

- What impact has the fast had on you as a member of a religious community?
- How important is it to you to attend religious community meetings? *Why? Frequency, who does this "community" consist of?*
- Could you describe for me what parts of fasting are more than just abstaining from food and liquids? *Experience, feeling, prescription*
- What meaning does a precept of your religion, such as that of fasting once a year, have for you?
- Can you assess whether it would be equally possible for you as a Bahá'í to experience yourself as a religious person if such religious rules did not exist?

- After fasting, was there anything you wanted to keep for the long term? How much have you been able to fulfill that resolution so far?
- What do you need/would you need to be able to keep those resolutions?
- Is there anything else you would like to add?
